# Supplementary material for: The covariance environment defines cellular niches for spatial inference
Source: bioRxiv. 2023 Apr 20:2023.04.18.537375. Preprint. [Version 1] doi: 10.1101/2023.04.18.537375 (PMC10153165; doi:10.1101/2023.04.18.537375)
Supplement: Supplement 2 [file NIHPP2023.04.18.537375v1-supplement-2.pdf]

bioRxiv preprint doi: https://doi.org/10.1101/2023.04.18.537375; this version posted April 20, 2023. The copyright holder for this preprint (which was not certified by peer review) is the author/funder, who has granted bioRxiv a license to display the preprint in perpetuity. It is made available under aCC-BY 4.0 International license.

1517 # SUPPLEMENTARY FIGURES

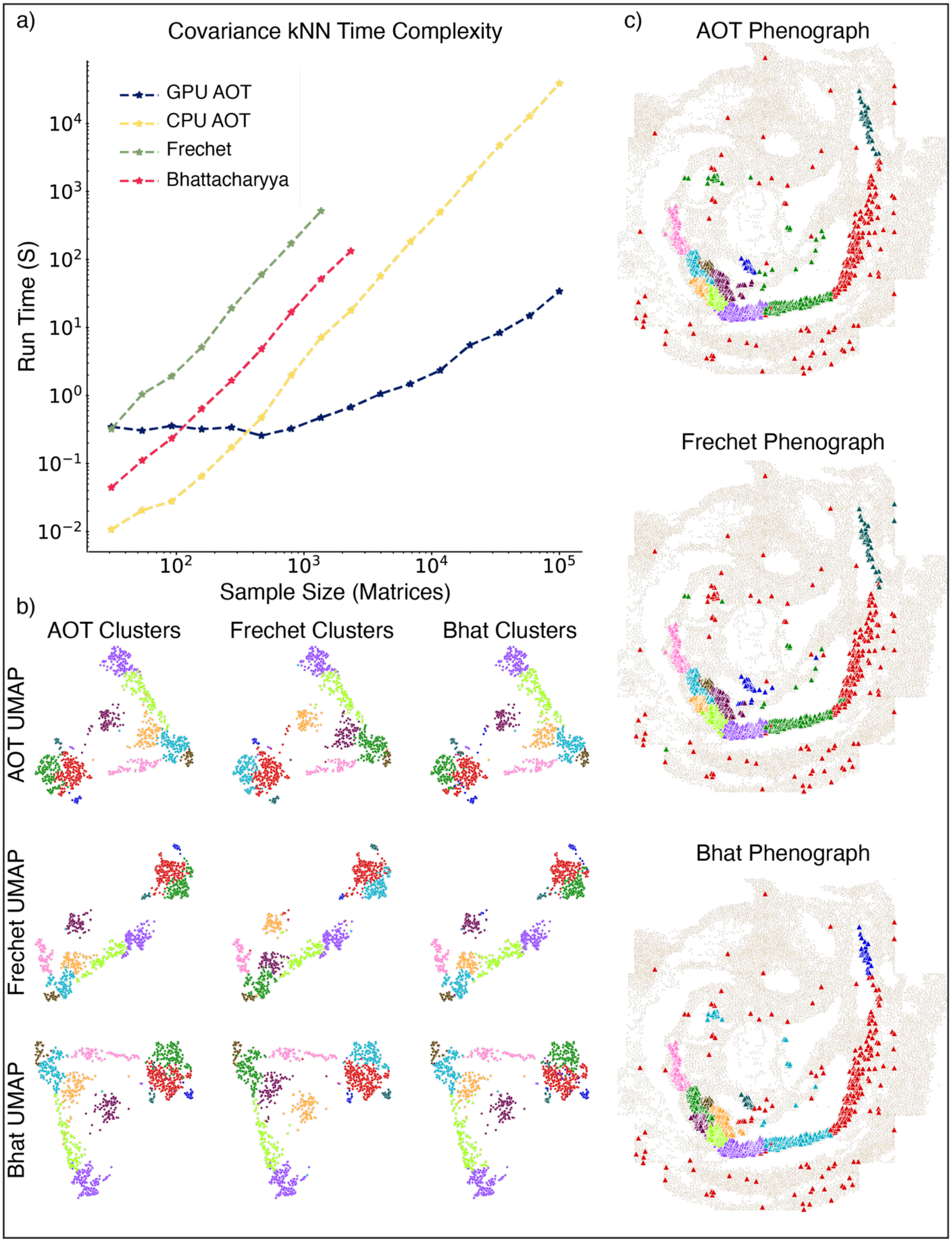

1518

bioRxiv preprint doi: https://doi.org/10.1101/2023.04.18.537375; this version posted April 20, 2023. The copyright holder for this preprint (which was not certified by peer review) is the author/funder, who has granted bioRxiv a license to display the preprint in perpetuity. It is made available under aCC-BY 4.0 International license.

Supplementary Figure 1. Approximate optimal transport (AOT) yields similar results to optimal transport and Bhattacharyya distance, but more efficiently. a, Run times for computing the kNN graph between sets of randomly generated covariance matrices at various sample sizes. Fréchet and Bhattacharyya run times are not shown for samples larger than 4,000 cells due to out-of-memory error on a 768-GB, 64-core computing cluster. **b**, COVET UMAP embeddings and PhenoGraph clustering of seqFISH splanchnic mesoderm by different metrics, colored by PhenoGraph clusters of each. **c**, seqFISH data from splanchnic mesoderm, colored by PhenoGraph clustering of COVET matrices according to each distance metric. Bhat, Bhattacharyya.

bioRxiv preprint doi: https://doi.org/10.1101/2023.04.18.537375; this version posted April 20, 2023. The copyright holder for this preprint (which was not certified by peer review) is the author/funder, who has granted bioRxiv a license to display the preprint in perpetuity. It is made available under aCC-BY 4.0 International license.

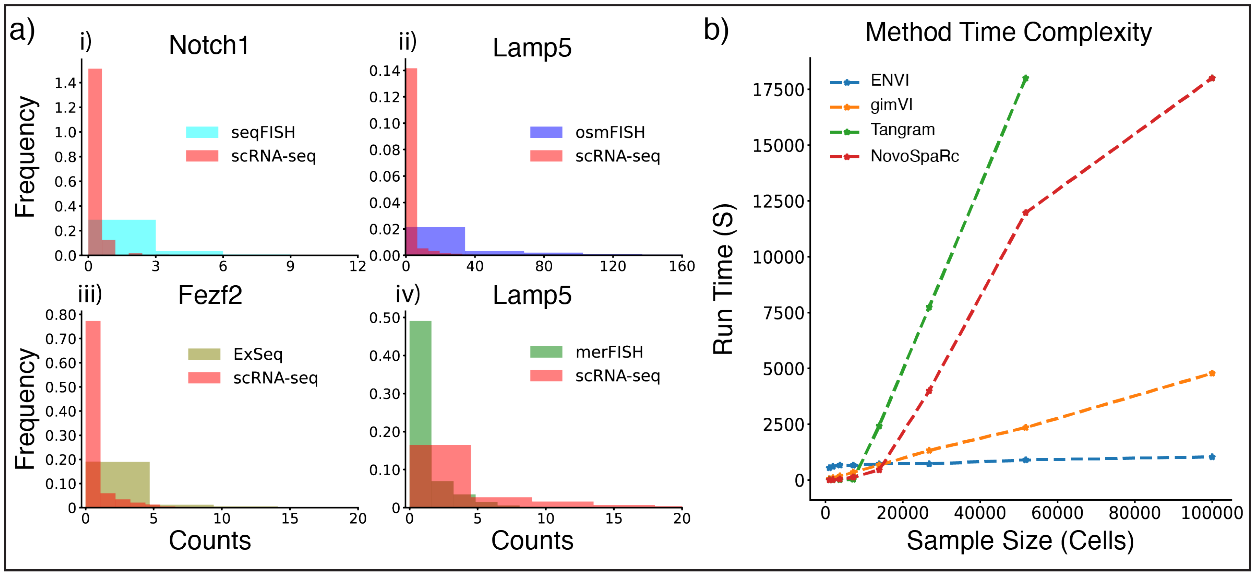

Supplementary Figure 2. Differences between spatial and scRNA-seq data and run time for different integration methods. a, The expression of three genes in four spatial datasets exhibits very different distributions from their complementary scRNA-seq data. **b**, Run time of ENVI, gimVI, Tangram and NovoSpaRc on integrating simulated scRNA-seq and spatial datasets of growing sizes. Run time for each method was capped at 5 hours (18,000 seconds) before the program was manually stopped.

bioRxiv preprint doi: https://doi.org/10.1101/2023.04.18.537375; this version posted April 20, 2023. The copyright holder for this preprint (which was not certified by peer review) is the author/funder, who has granted bioRxiv a license to display the preprint in perpetuity. It is made available under aCC-BY 4.0 International license.

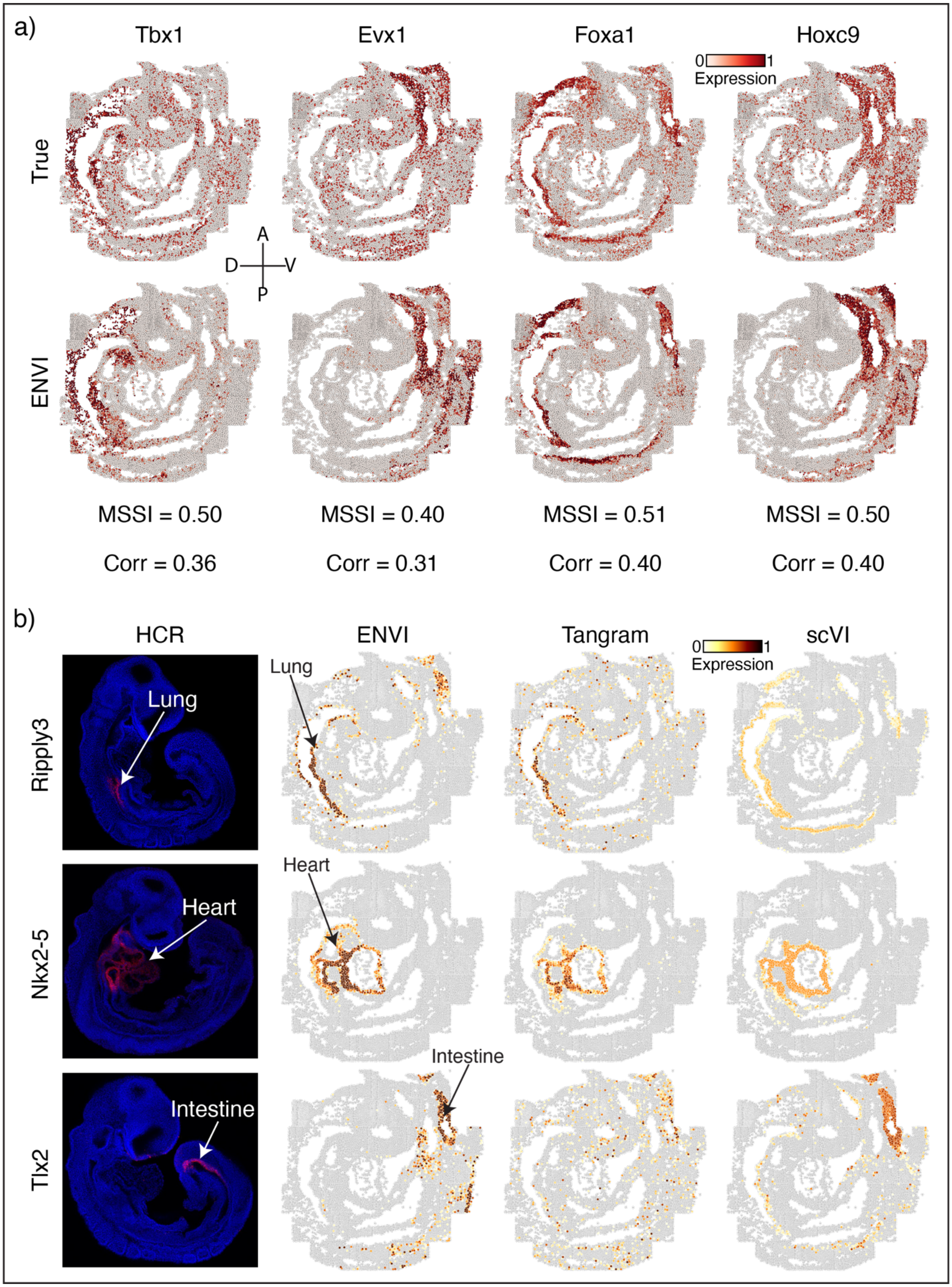

1535

bioRxiv preprint doi: https://doi.org/10.1101/2023.04.18.537375; this version posted April 20, 2023. The copyright holder for this preprint (which was not certified by peer review) is the author/funder, who has granted bioRxiv a license to display the preprint in perpetuity. It is made available under aCC-BY 4.0 International license.

Supplementary Figure 3. ENVI more accurately infers embryogenesis genes missing from a seqFISH panel compared to other methods. a, Imputed expression of withheld genes from the embryogenesis dataset (bottom) compared to true (measured) expression (top), with corresponding MSSI and Pearson correlation reconstruction scores below. **b**, HCR images of *Ripply3*, *Nkx2-5* and *Tlx2* and their imputation values according to ENVI, Tangram and gimVI. Organs marked by each gene are noted on the HCR and seqFISH images.

bioRxiv preprint doi: https://doi.org/10.1101/2023.04.18.537375; this version posted April 20, 2023. The copyright holder for this preprint (which was not certified by peer review) is the author/funder, who has granted bioRxiv a license to display the preprint in perpetuity. It is made available under aCC-BY 4.0 International license.

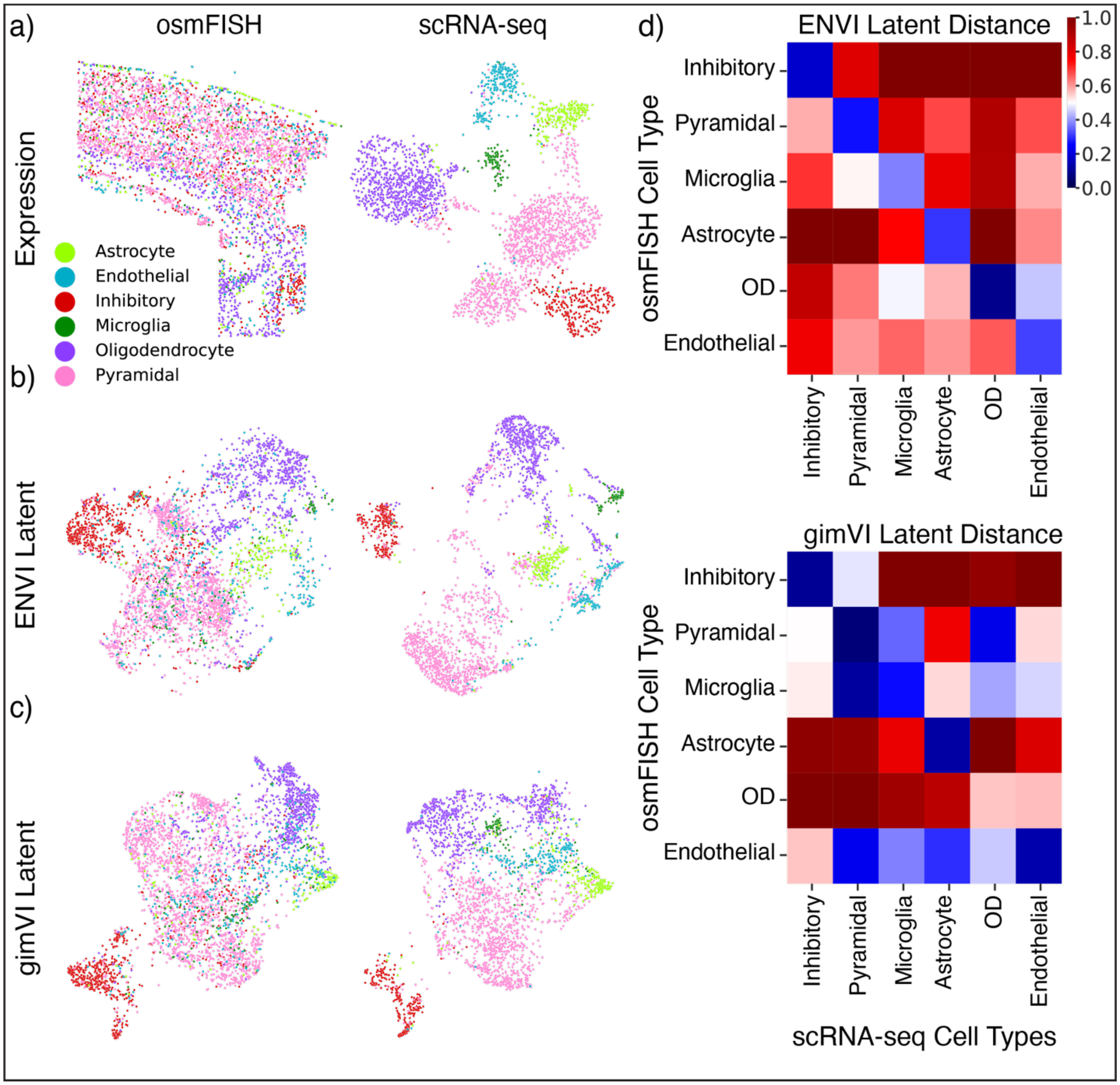

Supplementary Figure 4. ENVI can integrate between a 33 gene osmFISH panel and scRNA-seq assay of the somatosensory cortex. **a**, osmFISH with segmented cells and UMAP visualization of scRNA-seq datasets of the mouse somatosensory cortex, colored by cell types as annotated in Codeluppi et al[3]. **b**, UMAP visualizations of the ENVI integrated latent embedding of the osmFISH and scRNA-seq modalities, colored by cell types as in **a**. **c**, Same as **b**, but with latent embeddings from gimVI. **d**, Normalized distance between the center-of-mass of each cell type according to the ENVI and gimVI latent embeddings.

bioRxiv preprint doi: https://doi.org/10.1101/2023.04.18.537375; this version posted April 20, 2023. The copyright holder for this preprint (which was not certified by peer review) is the author/funder, who has granted bioRxiv a license to display the preprint in perpetuity. It is made available under aCC-BY 4.0 International license.

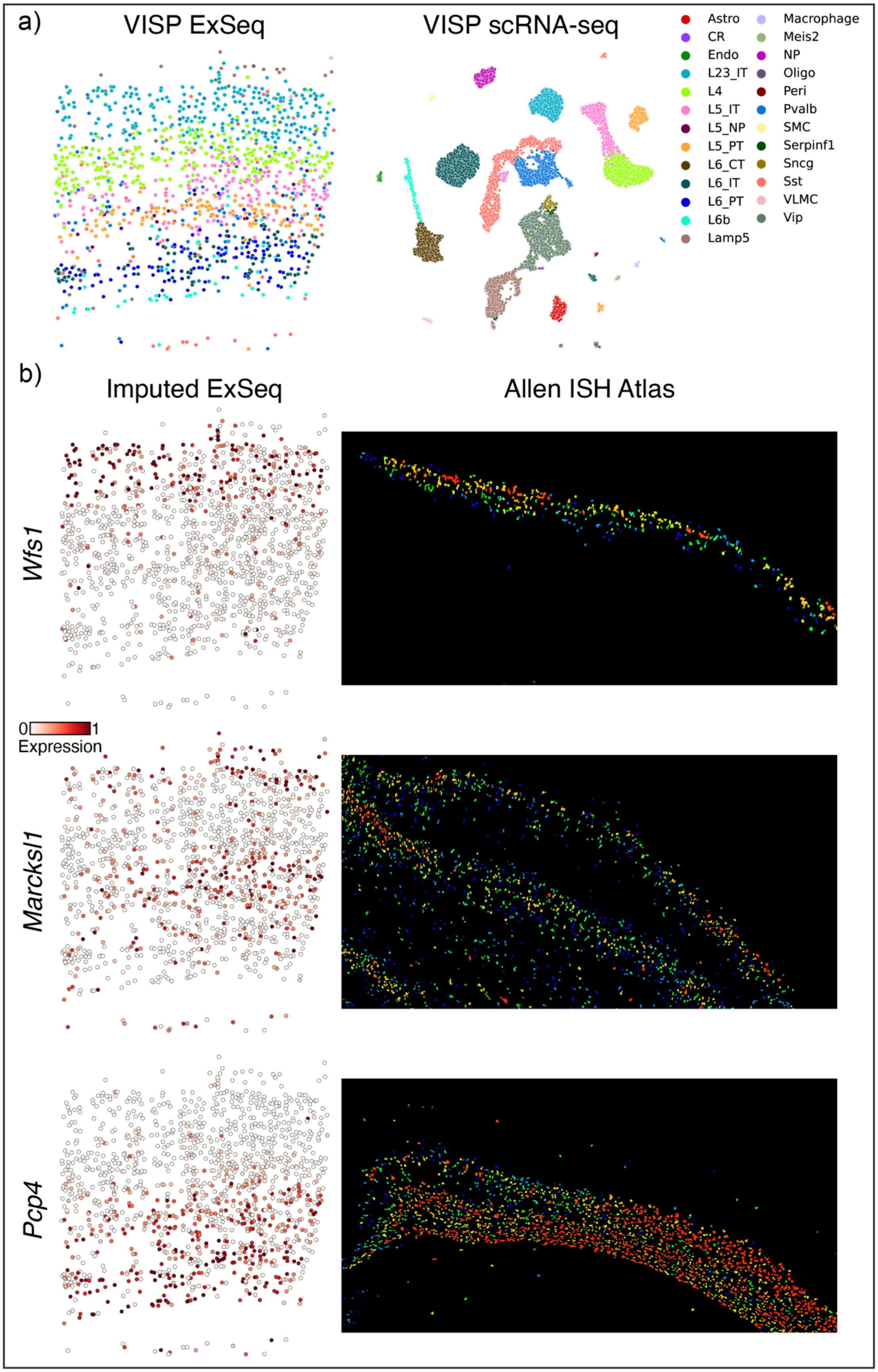

1552

bioRxiv preprint doi: https://doi.org/10.1101/2023.04.18.537375; this version posted April 20, 2023. The copyright holder for this preprint (which was not certified by peer review) is the author/funder, who has granted bioRxiv a license to display the preprint in perpetuity. It is made available under aCC-BY 4.0 International license.

Supplementary Figure 5. ENVI generalizes to imaging technologies beyond in situ hybridization (ISH). **a**, Spatial ExSeq (VISP[37]) data of the visual cortex (left) and the complementary scRNA-seq data, visualized by UMAP (right), colored into cell types. **b**, ENVI-imputed expression of the unimaged cortical markers *Wfs1*, *Marcksl1*, and *Pcp4* (left) and ISH imaging of the visual cortex from the Allen Brain Atlas (mouse.brain-map.org) (right).

bioRxiv preprint doi: https://doi.org/10.1101/2023.04.18.537375; this version posted April 20, 2023. The copyright holder for this preprint (which was not certified by peer review) is the author/funder, who has granted bioRxiv a license to display the preprint in perpetuity. It is made available under aCC-BY 4.0 International license.

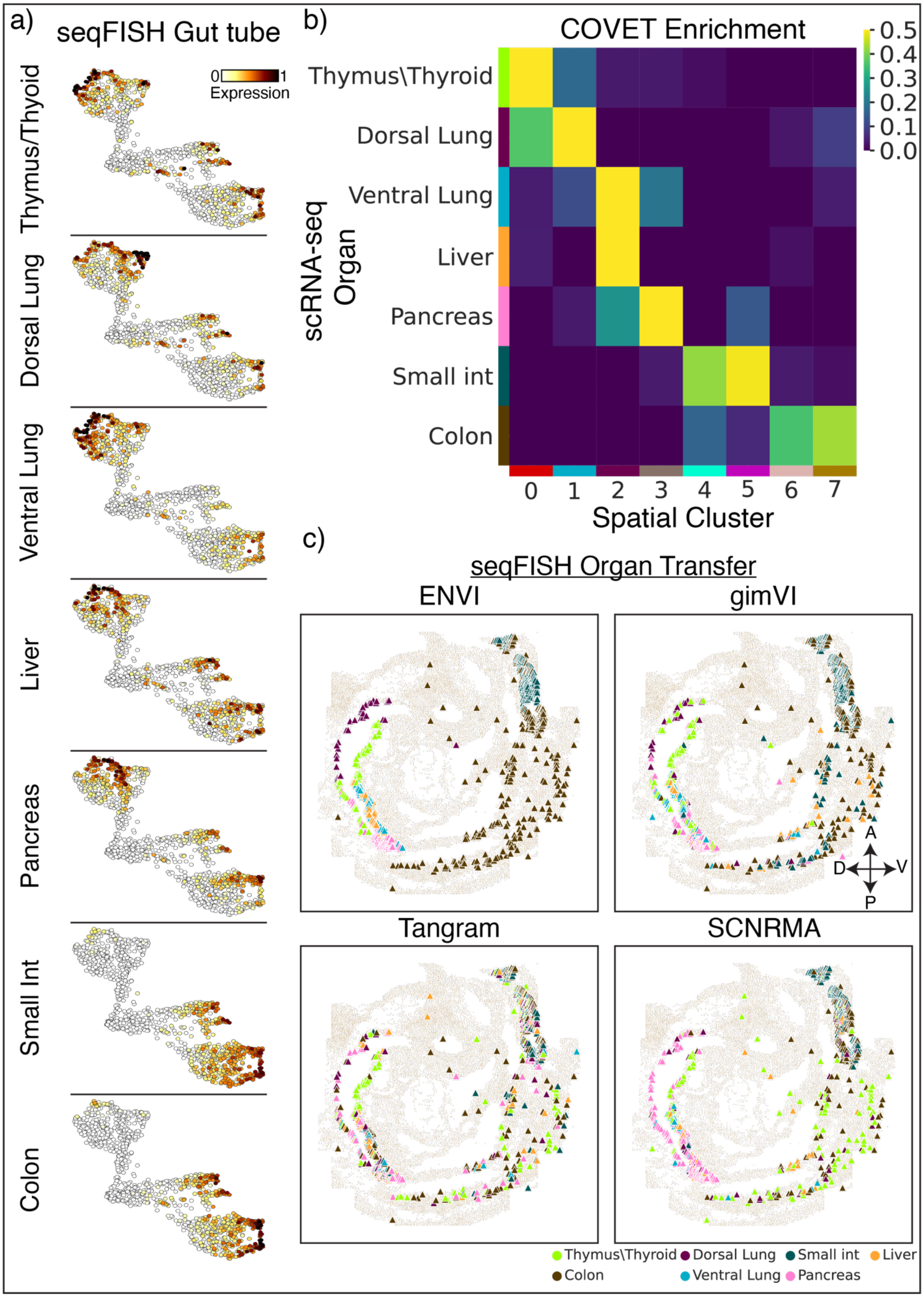

1558

bioRxiv preprint doi: https://doi.org/10.1101/2023.04.18.537375; this version posted April 20, 2023. The copyright holder for this preprint (which was not certified by peer review) is the author/funder, who has granted bioRxiv a license to display the preprint in perpetuity. It is made available under aCC-BY 4.0 International license.

Supplementary Figure 6. Spatial orientation of gastrulation from ENVI. a, UMAP of gut tube cells from seqFISH expression data[35], colored by average expression of reference gene sets. **b**, Proportion of scRNA-seq gut tube cells in each organ (row) which fall in each COVET cluster (columns). **c**. Assignment of developing organs to seqFISH gut tube cells via ENVI COVET space, gimVI latent space, Tangram cell-type mapping and Scanorama 'integrated' expression profiles, using the scRNA-seq labeled gut as reference.

bioRxiv preprint doi: https://doi.org/10.1101/2023.04.18.537375; this version posted April 20, 2023. The copyright holder for this preprint (which was not certified by peer review) is the author/funder, who has granted bioRxiv a license to display the preprint in perpetuity. It is made available under aCC-BY 4.0 International license.

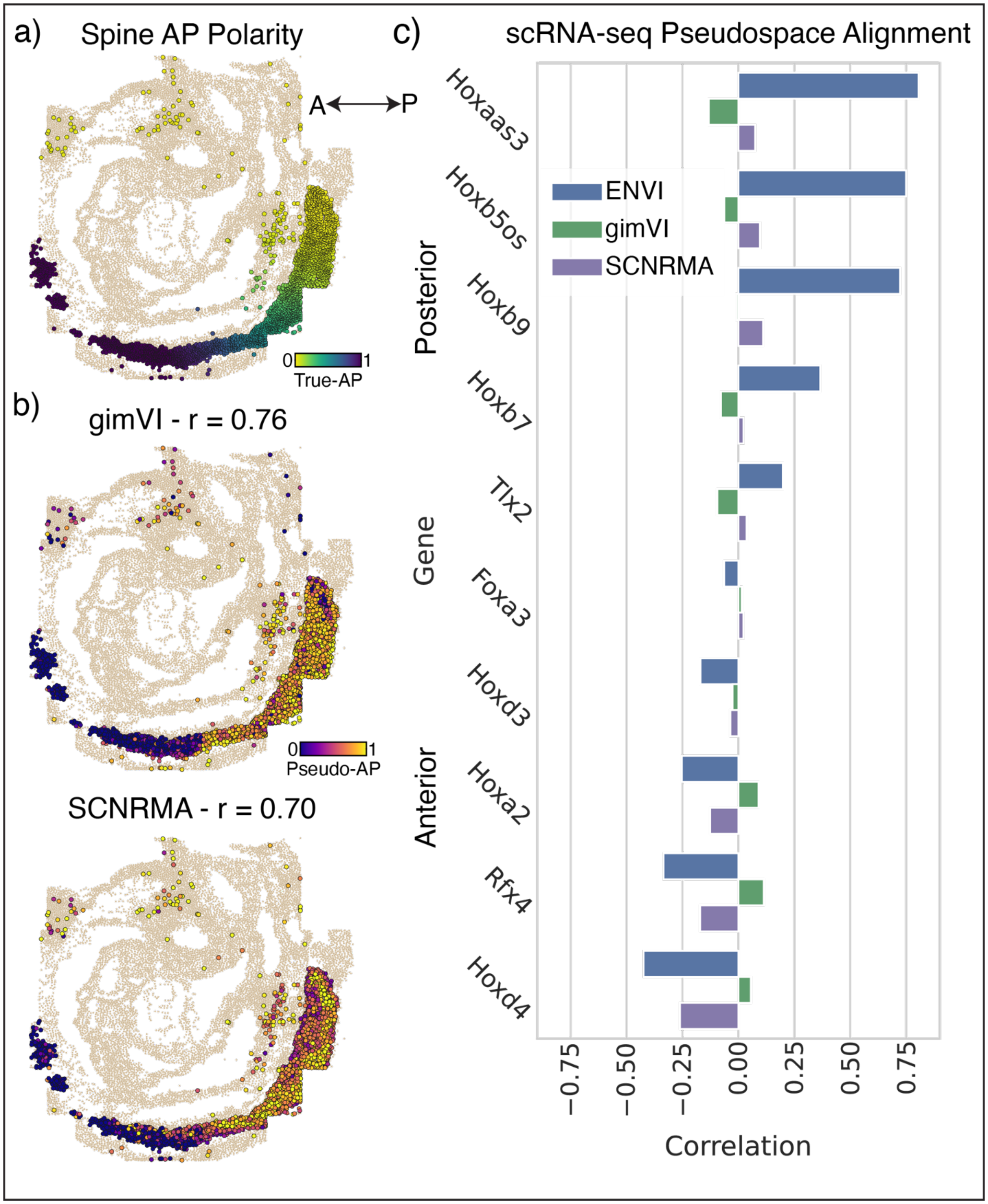

Supplementary Figure 7. ENVI can reliably recover the AP axis during spine development. **a**, Spine and NMP cells from seqFISH data, colored by AP polarity calculated from the first DC of their spatial coordinates. **b**, Pseudo-AP of seqFISH spine and NMP cells from DC analysis of gimVI and Scanorama. Values denote the Pearson correlation with the true AP axis. **c**, Pearson correlation of ENVI COVET, gimVI and Scanorama pseudo-AP of spine and

bioRxiv preprint doi: https://doi.org/10.1101/2023.04.18.537375; this version posted April 20, 2023. The copyright holder for this preprint (which was not certified by peer review) is the author/funder, who has granted bioRxiv a license to display the preprint in perpetuity. It is made available under aCC-BY 4.0 International license.

NMP scRNA-seq cells, for five canonical posterior markers (higher is better) and anterior markers (lower is better). Pseudo-AP axis is based on the diffusion component best aligned with true depth (DC 1, 2 and 3 for ENVI, gimVI and Scanorama, respectively).

bioRxiv preprint doi: https://doi.org/10.1101/2023.04.18.537375; this version posted April 20, 2023. The copyright holder for this preprint (which was not certified by peer review) is the author/funder, who has granted bioRxiv a license to display the preprint in perpetuity. It is made available under aCC-BY 4.0 International license.

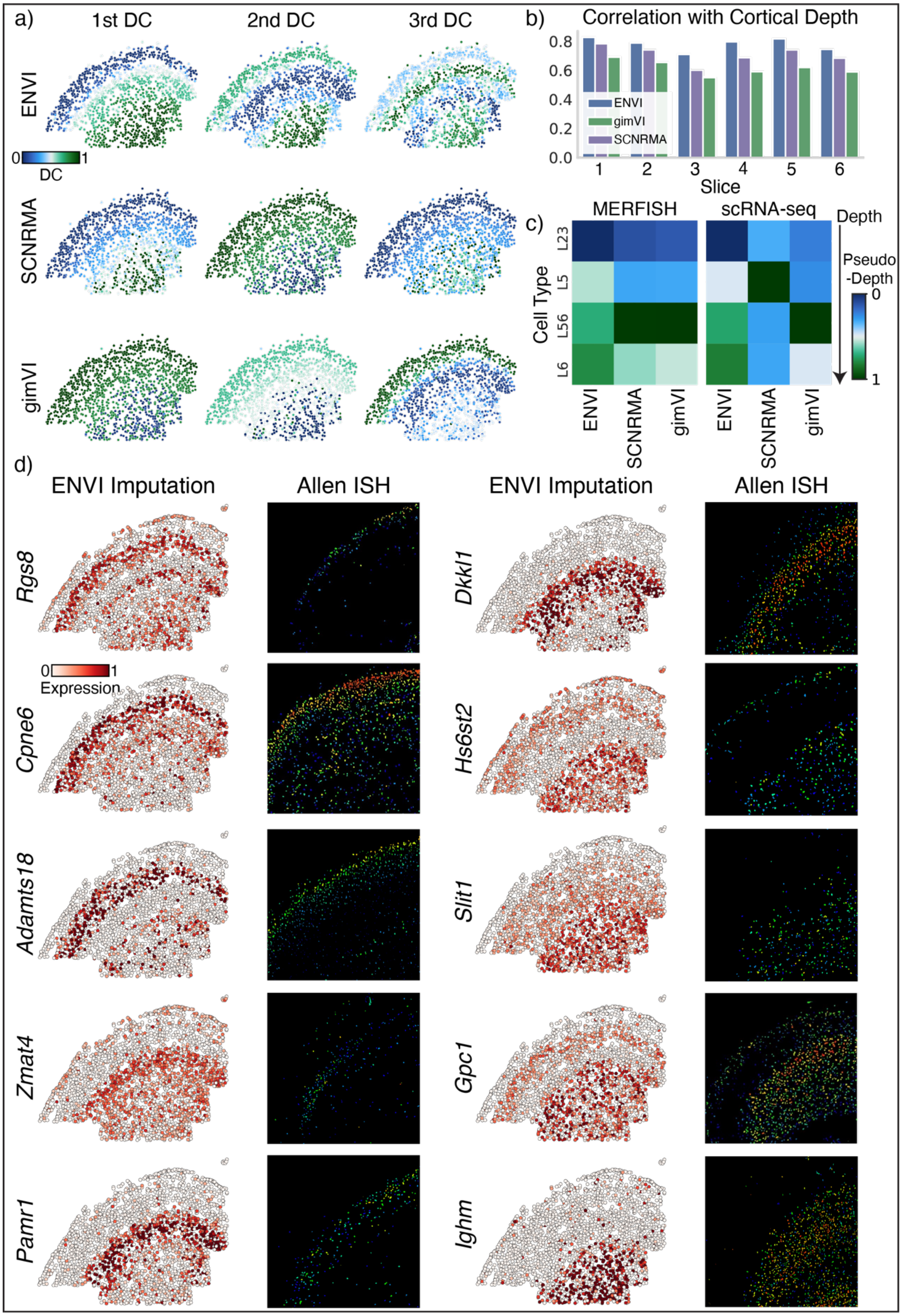

1574

bioRxiv preprint doi: https://doi.org/10.1101/2023.04.18.537375; this version posted April 20, 2023. The copyright holder for this preprint (which was not certified by peer review) is the author/funder, who has granted bioRxiv a license to display the preprint in perpetuity. It is made available under aCC-BY 4.0 International license.

Supplementary Figure 8. ENVI extends MERFISH panels to thousands of genes and predicts cortical depth of disassociated data. **a**, Glutamatergic neurons from the MERFISH motor cortex dataset[38], colored by the top three DCs from ENVI COVET, gimVI and Scanorama (SCNRMA). DC analysis for all methods was performed on the MERFISH and scRNA-seq co-embedding. **b**, Quantitative comparison of methods. Pearson correlation of pseudodepth with true cortical depth for glutamatergic neurons for each tissue slice in the MERFISH dataset. True depth for each slice was defined as the second DC of the spatial coordinates of the cells. Pseudo-Depth of each method was chosen as the DC most aligned with true depth (1st DC for all methods). **c**, Average z-scored pseudodepth according to each integration method of the glutamatergic neuronal subtypes, grouped by cortical layer. **d**, ENVI imputation of genes highlighted in **Fig. 6d**, and projected onto the MERFISH data, with corresponding ISH expression within the motor cortex from the Allen Brain Atlas (mouse.brain-map.org).
